# Comparison of crystalloid resuscitation fluids for treatment of acute brain injury: a clinical and pre-clinical systematic review and network meta-analysis protocol

**DOI:** 10.1186/s13643-018-0790-x

**Published:** 2018-08-17

**Authors:** Mary Thompson, Lauralyn McIntyre, Brian Hutton, Alexandre Tran, Dianna Wolfe, Jamie Hutchison, Dean Fergusson, Alexis F. Turgeon, Shane W. English

**Affiliations:** 1 Clinical Epidemiology Program (CEP), Ottawa Hospital Research Institute, Ottawa, Ontario Canada; 2 Department of Medicine (Critical Care), University of Ottawa, CPCR Building, 501 Smyth Rd, CPCR Box 201B, Ottawa, Ontario K1H 8L6 Canada; 3 School of Epidemiology and Public Health, University of Ottawa, Ottawa, Ontario Canada; 4 Department of General Surgery, University of Ottawa, Ottawa, Ontario Canada; 5 Department of Critical Care, Sick Kids Hospital, Toronto, Ontario Canada; 6 Department of Anesthesiology and Critical Care Medicine, Division of Critical Care Medicine, Université Laval, Quebec City, Quebec Canada; 7 CHU de Québec - Université Laval Research Center, Population Health and Optimal Health Practices Unit (Trauma - Emergency - Critical Care Medicine), CHU de Québec, Université Laval (Hôpital de L’Enfant-Jésus), Quebec City, Quebec Canada

**Keywords:** Systematic review, Meta-analysis, Brain injury, Resuscitation, Crystalloids

## Abstract

**Background:**

Current guidelines identify the choice of fluid resuscitation as important in minimizing the incidence of secondary brain injury from cerebral edema. It is widely accepted that isotonic crystalloid resuscitation fluids, specifically normal saline (NS), are optimal for resuscitation and that other relatively hypotonic fluids, such as Ringer’s lactate (RL), should be avoided in this patient population. The aim of this review is to systematically compare the use of relatively hypotonic versus isotonic crystalloid resuscitation fluids in clinical and pre-clinical models of acute brain injury and their effect on outcomes. In recognition of the potential need for a network meta-analysis (NMA), we have also included all other relevant crystalloid resuscitation fluids as interventions of relevance to potentially inform indirect comparisons.

**Methods:**

Systematic searches of MEDLINE, Embase, and Web of Science BIOSIS Previews® will be used to identify eligible clinical and pre-clinical studies, which included studies examining acute brain injury (human and in vivo animal brain injury models) within the first 7 days of therapy. The intervention of interest is the intravenous use of relatively hypotonic crystalloid resuscitation fluids (e.g., Ringer’s lactate, Hartmann’s or Plasma Lyte® fluids). The main comparator of interest is an isotonic crystalloid resuscitation fluid, specifically normal saline (0.9%). Other crystalloid resuscitation fluids (e.g., hypertonic saline (3–23.4%)) will also be included as an additional intervention of interest. The primary outcome measures of interest are intracranial pressure (ICP) and cerebral perfusion pressure (CPP). Secondary outcomes include the effect of resuscitation on cerebral edema, brain and serum osmolarity, and electrolyte concentrations and clinical outcomes including modified Rankin Scale (mRS), (extended) Glasgow Outcome Scale (GOS/eGOS), and mortality. Separate meta-analyses will be conducted to quantify the effects of the different fluid resuscitation on acute brain injury outcomes in clinical and pre-clinical populations. Network meta-analyses to compare interventions will also be performed to compare the effects of different interventions.

**Discussion:**

This systematic review will comprehensively summarize the difference in treatment efficacy of various crystalloid resuscitation fluids in acute brain injury. This review is essential to underscore the evidence, or lack thereof, present in the literature to date to support current preference-driven practice and to direct future study.

**Systematic review registration:**

PROSPERO #CRD42016042960

**Electronic supplementary material:**

The online version of this article (10.1186/s13643-018-0790-x) contains supplementary material, which is available to authorized users.

## Background

Acute brain injury is a global health issue responsible for significant death and disability worldwide with rising incidence [1]. It is associated with a complicated hospital course where the primary injury is commonly associated with further neurological damage, known as secondary brain injury, including ischemia, neuronal death cascades, cerebral edema, and inflammation [2–4]. Episodes of hypotension, which are common in traumatic brain injury patients, can further exacerbate secondary brain injury and thus increase the probability of a poor outcome [2]. As such, fluid resuscitation, a common intervention in acute medicine, is often utilized to combat hypotension and maintain adequate cerebral perfusion. Given the numerous fluid compositions available for resuscitation, the selection should theoretically be based on physiological principles related to the unique function of the affected organ and/or system. However, significant controversy exists surrounding which is the optimal fluid in critically ill patients. Thus, it is clinician preference that mainly dictates clinical practice, with considerable variation between institutions and specialties [5]. Efforts to resolve this discrepancy have been made in many areas of critical care research, including a systematic review comparing the use of crystalloid and colloid fluids for resuscitation in septic shock [6]. Dissimilarly, there is remarkably little research related to the study of these fluids for the treatment of acute brain injury. Existing work demonstrates that metabolic derangements are common and can further lead to secondary brain injury [7]. Whether this is related to the consequence of fluid administration or the primary injury itself remains unclear. Similar findings are seen in subarachnoid hemorrhage [8]. Management of such injuries thus presents a significant challenge to critical care medicine.

Current guidelines identified the choice of fluid resuscitation as an important initial step in minimizing the incidence of secondary brain injury from cerebral edema [9]. These recommendations are vague with regard to specific fluid choice [9]. However, 0.9% normal saline (NS) is the most commonly used fluid for resuscitation in patients with acute brain injury as it is the prototypical “isotonic” solution relative to plasma [2, 10]. Other fluids with lower sodium concentrations, such as Ringer’s lactate (RL) solution, are thus considered hypotonic when compared to plasma and are avoided [2, 10].

To date, no large randomized controlled trials have been conducted comparing Ringer’s lactate to normal saline directly in an acutely brain injured population; therefore, there is no existing data to provide conclusive evidence as to which crystalloid resuscitation fluid confers a greater benefit in acute treatment. Furthermore, recent practice management guidelines concluded that there was in fact insufficient evidence to recommend one crystalloid resuscitation fluid over another in the acute pre-hospital fluid resuscitation of injured patients [11]. Given this expected lack of direct evidence and the availability of multiple key comparators, a network meta-analysis (NMA) may be necessary to inform this comparison. NMA is an extension of traditional pairwise meta-analysis enabling the comparison of multiple interventions based on available direct and indirect evidence [12, 13]. Our systematic review will synthesize the existing knowledge from published clinical and pre-clinical studies examining the use of relatively hypotonic versus isotonic crystalloid resuscitation fluids for the treatment of acutely brain-injured patients and their effect on a variety of physiological and clinical outcomes.

## Objectives

**Fig. 1:**
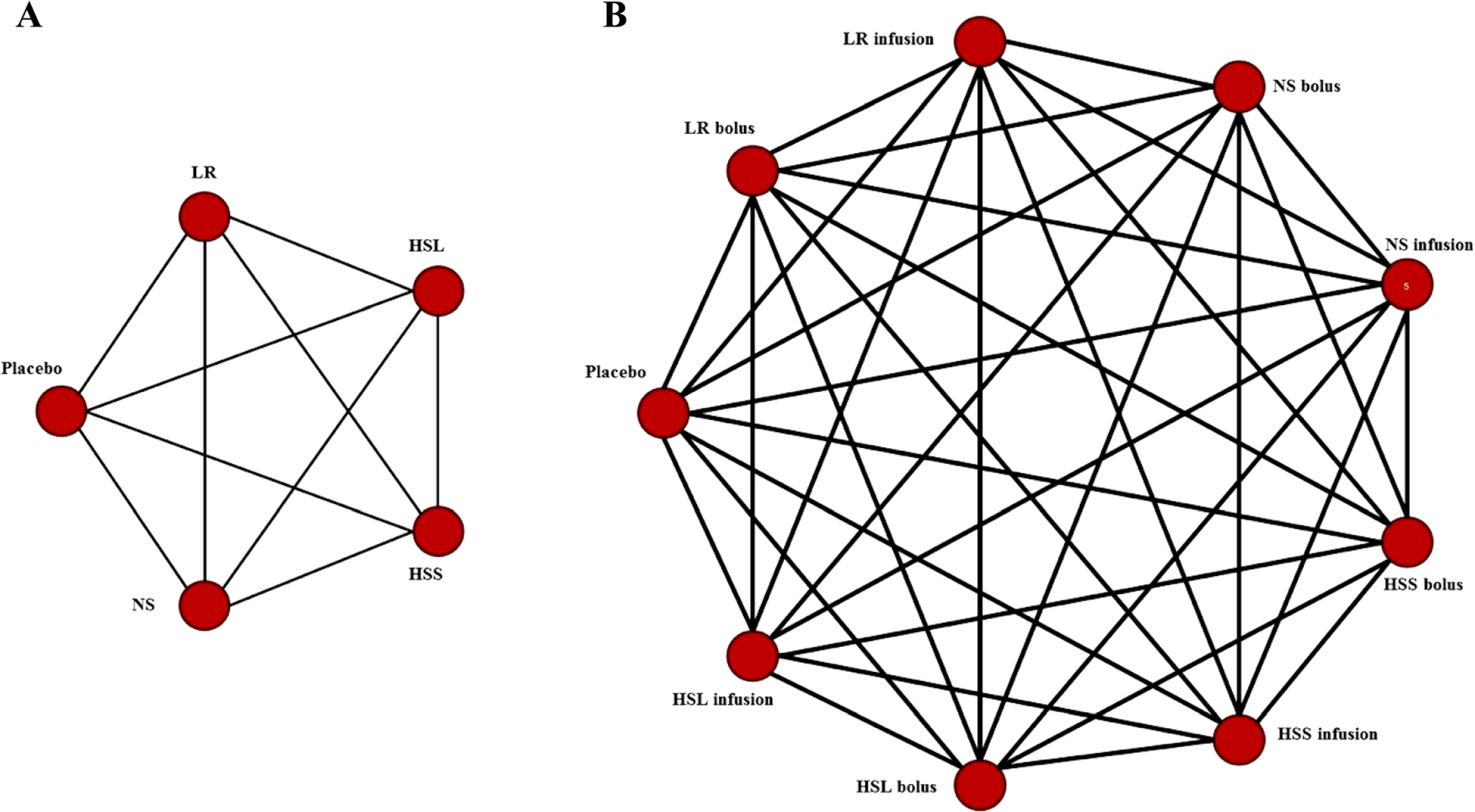
Sample NMA maps. **a** Simple NMA map wherein the interventions of interest are considered at the treatment level only, without consideration of method of administration. **b** Dynamic NMA map wherein the interventions of interest are considered at both the treatment and method of administration levels simultaneously. HSL, hypertonic sodium lactate; HSS, hypertonic saline solution; LR, Ringer’s lactate; NS, normal saline

The primary objective of this review is to compare the effect of relatively hypotonic versus isotonic crystalloid resuscitation fluids (i.e., Ringer’s lactate, Hartmann’s, and Plasma Lyte® versus normal saline (0.9%)) for resuscitation in early acute brain injury (first 7 days post-injury) on intracranial pressure (ICP) and cerebral perfusion pressure (CPP). In anticipation of a lack of direct evidence, we will also consider hypertonic crystalloid fluids (i.e., 3–23.4% hypertonic saline) as a third intervention of interest with an eye toward the need for indirect comparison methods (such as adjusted indirect comparisons or network meta-analysis; see Fig. 1). We have selected ICP/CPP as a primary outcome measure as it is objective, relatively easily, and frequently measured and has clinical sensibility in the possible biologic effects of these fluids. Although ICP and CPP are traditionally measured in traumatic brain injury populations, and thus may limit generalizations of the results to other “non-monitored” acute brain injury populations, their use in these other populations has been increasing [14]. Secondary objectives include the effect of these different solutions on (1) cerebral edema, (2) serum and brain electrolyte concentrations (Na+, Cl−) and osmolality, and (3) clinical outcomes including modified Rankin Scale (mRS), Glasgow Outcome Scale (GOS), extended Glasgow Outcome Scale (eGOS), and mortality. Finally, as tertiary outcomes, we will report on any adverse outcomes as described in the primary citations (e.g., ARDS and AKI incidence).

## Methods/design

This review will be conducted in accordance with The Cochrane Collaboration [15] principles for Systematic Reviews and reported following the PRISMA guidelines [16]. This protocol was drafted in accordance with PRISMA-P guidelines (see checklist in Additional file 1) and has been registered with the PROSPERO International Prospective Register of Systematic Reviews (#CRD42016042960).

### Search strategy and data sources

Our search strategy will be conducted using Epub Ahead of Print, In-Process & Other Non-Indexed Citations, Ovid MEDLINE(R) Daily and Ovid MEDLINE(R), EMBASE Classic + Embase, Web of Science BIOSIS Previews® and EBM Reviews (including Cochrane Central databases) from inception to the moment of review. EMBASE also includes the abstract publications from major international conferences including the International Stroke Conference, Neurocritical Care Society Meeting, Society of Critical Care Medicine, and the International Symposium on Intensive Care and Emergency Medicine. A comprehensive search strategy will be constructed and implemented by a health information specialist with systematic review experience, in collaboration with the research team. MeSH terms will be used to capture each of the principal elements of the research question. We will restrict our strategy to focus on population (acute brain injury) and intervention/exposure (hypotonic crystalloid solutions and hypertonic solution alternatives—see Eligibility criteria) and will not be limited by outcome studied to increase our yield. A sample search strategy is presented in Additional file 2. Upon completion, identified citations will be exported to a cloud-based citation manager (DistillerSR v2: Systematic Review and Literature Review Software) for study selection. Manual review of the reference lists of all included studies and previous systematic reviews will be conducted. A final gray literature search will be conducted using “Google Scholar” as well as a review of the trial register (clinicaltrials.gov, WHO ICTRP) for any ongoing and unpublished studies. Duplicate citations will be removed. The search strategies will be kept up to date to the time of the end of the review.

### Study eligibility

For the purposes of this review, we will refer to normal saline as an isotonic solution and those with lower sodium concentrations (e.g. Ringer’s Lactate, Plasma Lyte®) will be referred to as “relatively hypotonic.” Those crystalloid solutions with higher sodium concentrations (e.g., 3% saline) will be referred to as hypertonic. We will include pre-clinical studies that compare a relatively hypotonic crystalloid resuscitation fluid (Ringers Lactate, Hartmann’s or Plasma Lyte®) to an isotonic (normal saline (0.9%)) or hypertonic (i.e., hypertonic saline (3–23.4%)) crystalloid resuscitation fluid. No overtly hypotonic solution (e.g., 5% dextrose in water, 5% in 0.45% saline) or colloid solutions will be included given their very different physiological properties that would have them behave significantly differently as compared to crystalloid solutions of interest in this review.

For clinical studies, we will include observational studies that have a control group for comparison, whether prospectively or retrospectively conducted, and intervention studies (e.g., randomized controlled trials). An iterative process for study selection will be followed using the criteria set out in Table 1.

**Table 1:** Eligibility criteria for the systematic review (expanded detail provided in Additional file 3)

Criteria	Description
Population	We will include all studies examining acute brain injury (human and in vivo animal brain injury models) with treatment initiation within the first 7 days post-injury. We are targeting acute primary neurological diagnoses such as traumatic brain injury, stroke, hemorrhage, or post-neurosurgical care.
Intervention	The intervention of interest is the intravenous use of *relatively hypotonic* crystalloid resuscitation fluids. These include Ringer’s lactate, Hartmann’s or Plasma Lyte® fluids. These can be administered as bolus or maintenance infusions. There will be no limits on dose or frequency of administration.
Comparator(s)	The main comparator of interest is an isotonic crystalloid resuscitation fluid, specifically normal saline (0.9%), which will serve as the prototypical control. Other crystalloid resuscitation fluids (e.g., hypertonic saline (3–23.4%)) will also be included as an additional comparator of interest. These can be administered as bolus or maintenance infusions. There will be no limits on dose or frequency of administration.
Outcome(s)	The primary outcomes of interest are: • Intracranial pressure (ICP; mmHg) and cerebral perfusion pressure (CPP; mmHg) Secondary outcomes include: • Cerebral edema (mL H_2_O/g dry), serum and/or brain electrolyte concentrations (Na+, Cl−; mmol/L) and osmolarity (mOsmol/L [29]), clinical outcomes as assessed by any of modified Rankin Scale (mRS), Glasgow Outcome Scale (GOS), extended Glasgow Outcome Scale (eGOS), mortality; and Tertiary outcomes include: • Any adverse events as defined by the study authors
Study design	We will include all completed publications reporting the intravenous use of crystalloid fluids in acute brain injury. • Clinical: randomized controlled trials, quasi-randomized trials, and retrospective and prospective studies that include a control group for comparison • Pre-clinical: randomized laboratory studies There will be no date or language restrictions applied. In-progress studies and letters to the editor identified will be included in a qualitative analysis. Case reports, case series, editorial reviews, and guidelines will be excluded.

### Study selection process

All records will first be screened by title and abstract. All citations clearly not relevant to the review (e.g., wrong population and narrative review) will be excluded. This process will be performed in duplicate by two independent reviewers. Any citation in which an abstract is not available and where suitability for inclusion is questioned will proceed to the next stage. All citations not excluded in the first screen will have full articles retrieved for a second review, in duplicate by independent reviewers, and the selection criteria applied. Any differences in classification between the two independent reviewers will be reviewed and consensus decision made. A third independent senior reviewer will be used in any instance in which consensus is not reached.

### Data extraction

A data extraction form will be prepared a priori in MS Excel (Microsoft Corporation, Seattle, Washington) and piloted prior to duplicate extraction by two independent reviewers. The data extraction form will be designed to capture information regarding study characteristics, design, and methods (i.e., title, authors, journal/source, year of publication, country, type of study, study period, total number of subjects, case ascertainment and/or inclusion/exclusion criteria, randomization, allocation concealment and blinding methods (where applicable)); study population characteristics (i.e., clinical studies: age, sex, primary neurological diagnosis, injury severity/characteristics, comorbidities; pre-clinical studies: species, species detail, age, sex, weight, model type and experimental conditions, injury severity/characteristics); interventions and co-interventions [crystalloid resuscitation fluid type, dose, frequency, adjunctive fluid administration, use of a management protocol, use of co-interventions for intracranial pressure management strategies (including hyperosmolar therapy, paralysis, hyperventilation, barbiturate coma, decompression)]; and outcome (our pre-defined primary, secondary and tertiary outcomes).

Outcome data will be extracted based on a priori specified time points and dosage format. Given fluid resuscitation is the intervention of interest, data will be extracted for early (0–6 h; at hourly time points) and delayed resuscitation (up to 7 days post-injury; at 12, 24, 48, etc. hours) in order to evaluate both the immediate and delayed effects of treatment. Data will then be pooled by dosage format [means of administration (bolus vs maintenance infusion) and total dose] for the purposes of analysis.

Disagreements in data extraction will be resolved by consensus or by a third reviewer with methodologic and clinical expertise as required. If there are missing data, the corresponding authors of the studies will be contacted and if the data is not located, the remaining available data will be analyzed. The possible impact of the missing data will then be discussed as a limitation.

### Risk of bias

Risk of bias will be assessed using the Newcastle-Ottawa Scale [17] for observational studies, the Cochrane Collaboration tool for assessing the risk of bias in randomized controlled trials (RCTs) [18] and SYRCLE’s risk of bias tool for animal studies [19]. Bias risk assessment will be completed in a similar fashion as the study selection process: in duplicate by two independent assessors. Cases of discordance not resolved by consensus will be reviewed by a third senior assessor. Risk of bias assessment of all included studies will be summarized and presented in table format. Low risk of bias will be defined as those studies with score of ≥ 7 using the Newcastle-Ottawa Scale, those deemed low risk across all domains of the Cochrane Collaboration’s tool for assessing risk of bias, or those receiving an overall “yes” judgment across all signaling questions used in SYRCLE’s risk of bias tool. The authors recognize that no formal cut-offs exist to define low or high risk of bias with the Newcastle-Ottawa Scale; however, we deem that in order for low risk of bias to exist in an observational study, there must be excellent reporting, high internal and external validity with little risk of confounding such that high scores in each of these domains are necessary to meet low-risk criteria.

### Patient and public involvement

No patients were involved in the creation of this protocol.

## Analysis plan

A description of all included studies, including demographic, clinical and methodological quality, will be reported with the aid of tables and text. These characteristics will be studied and assessed by the research team in considering the validity of the transitivity assumption which underpins network meta-analysis [20]. As it is anticipated that some of the most relevant studies within the literature will be of an observational design and as such studies may be at particular risk of bias and confounding [21, 22], careful inspection of study reports will be performed to monitor for potential design limitations. We will follow a sequential approach to synthesis of the evidence whereby we will first consider RCT evidence alone, followed by subsequently including prospective and retrospective non-randomized evidence. We will also explore analyses using a hierarchical modeling approach as described elsewhere [22].

### Primary outcome

We anticipate that ICP and CPP will be presented in the majority of included studies as continuous data and described with means or medians and corresponding standard deviations or interquartile ranges (or ranges), respectively. Mean differences with 95% confidence intervals (CI) will be used to present and summarize continuous data; data reported as medians with interquartile ranges will be converted to means and standard deviations using methods outlined by Wan et al. [23]

### Secondary outcome

We anticipate that the secondary outcomes will be presented as a combination of continuous and dichotomous data. All continuous outcome variables, including brain/serum electrolyte concentrations and osmolarity, will be described and summarized in the same fashion as our primary outcomes. Cerebral edema, serum/brain electrolyte concentrations, and osmolarity will be reported in 6-h intervals up to 72 h post fluid administration. It is anticipated that clinical outcomes (mRS, GOS, and eGOS) will be presented as ordinal data, although the authors may have dichotomized it prior to publication. We will report clinical outcomes at discharge, 30 days, and at 3, 6, and/or 12 months. For studies that use these ordinal grading scale to describe outcome (e.g., mRS, GOS, eGOS), where possible, we will compare proportions in each category between the different groups. Dichotomous outcomes, such as adverse events, are expected to be presented as relative risk ratios (RR) or odds ratios (OR) with 95% CI in which data presented as a OR will be converted to RR where possible [24].

### Primary analyses

Separate analyses will be conducted for both clinical and pre-clinical data. Preliminary analyses will consist of traditional pairwise meta-analyses using random effects inverse variance models which will be carried out for each treatment comparison in evidence networks under study. Suitability for meta-analysis will be determined by the degree of heterogeneity (clinical and statistical) observed between the studies. The degree of statistical heterogeneity within each pairwise comparison will be assessed using the *I*^2^ measure, wherein values > 75% will be considered to be indicative of a high degree of heterogeneity. Dichotomous and continuous endpoints will be reported in terms of odds ratios and mean differences with 95% confidence intervals. If sufficient data exists and the included studies are considered of sufficient clinical and methodologic homogeneity to satisfy the transitivity assumption [20], Bayesian network meta-analyses will be conducted to calculate the effect of relative hypotonic fluids on each outcome of interest, based on well-established methods by the National Institute for Health and Care Excellence (NICE) [25–27].

In addition to data availability, we will assess the homogeneity of the evidence base, as well as consider statistical heterogeneity of the components of the network (as outlined above) in deciding whether to proceed with the performance of NMAs. Should NMAs be pursued, random effects (RE) NMAs will be performed using models for binomial and continuous endpoints (as appropriate) using existing models described elsewhere [25]. Mean differences and relative risk ratios will be reported to express differences between treatments for continuous and dichotomous endpoints, respectively, along with corresponding 95% credible intervals. Following unadjusted analyses and dependent upon the availability of study information, secondary analyses based upon subgroups or meta-regression methods will be considered to account for between-study differences related to animals enrolled (e.g., murine vs porcine models), models of disease induction (e.g., ischemia vs trauma), and considerations of housing and husbandry (e.g., co-housed vs isolated). We will also check for indications of the presence of inconsistency in the available data by fitting inconsistency models and reviewing scatterplots of deviance residuals [26]. Adequacy of model fit for NMAs will be assessed for all analyses by comparing the posterior residual deviance to the corresponding number of unconstrained data points (approximately equal if fit is adequate). Model convergence for NMAs performed will be assessed using trace plots, the Brooks-Gelman-Rubin statistic, and inspection of Monte Carlo errors. Three chains will be fitted in WinBUGS for each analysis, with a minimum of 50,000 iterations and a burn-in of at least 50,000 iterations.

For both approaches to meta-analysis, grouping of therapies will be performed using two strategies. A simpler representation will first be explored wherein the interventions of interest are considered at the treatment level only, without consideration of method of administration (see Fig. 1a). The second representation will consider both treatment and method of administration simultaneously (see Fig. 1b). Grouping of interventions will be discussed again amongst the research team prior to the start of data analyses.

All analyses will be conducted using R (R Core Team, Vienna, Austria) and WinBUGS software (MRC Biostatistics Unit, Cambridge, UK). NMAs will be based on the WinBUGS code freely available in the NICE Evidence Synthesis TSD Series [25–27]. Reporting of findings will be guided by the PRISMA Statement [16] and the PRISMA Extension Statement for Network Meta-Analysis, as appropriate [28].

### Subgroup and sensitivity analyses

In addition to the previously identified pre-planned subgroup and regression analyses, we will examine clinical heterogeneity with low vs large volume resuscitation groups. To test the robustness of our findings, we plan to pursue sensitivity analyses including only those studies judged to be at low risk of bias.

## Discussion

The results of this systematic review will systematically summarize the evidence available on the difference in treatment efficacy of various crystalloid resuscitation fluids in acute brain injury with a special interest in relatively hypotonic compared to isotonic crystalloid resuscitation fluids. These results are important since isotonic crystalloid resuscitation fluids, specifically NS, are considered the optimal fluid for resuscitation in such a population, despite an apparent absence of high-quality clinical evidence to support these recommendations. Therefore, this reflects a largely dogmatized notion founded in routine and clearly lacking in literary support. This systematic review is essential to understand the present evidence, or lack thereof, in the literature that supports current guidelines and recommendations while also highlighting areas in need of further study.

We anticipate that there will be a paucity of literature available on this subject with available evidence resulting from both clinical and pre-clinical studies. As such, we will implement a broad search of multiple databases to capture any and all studies relevant to our question. We will also keep our inclusion criteria broad, especially for title/abstract screening to ensure that we do not pre-emptively exclude any potentially relevant studies. Furthermore, given that we are unsure of what exists with regard to the literature on this subject, we chose to focus our search on RL in order to capture all studies using any other crystalloid resuscitation fluid either as comparator or control.

The application of network meta-analysis to studies in the pre-clinical literature, to our knowledge, is novel. We plan to empirically explore the synthesis of such data while considering the feasibility and importance of adjustments for several factors related to the types of animals studied, models of induction employed, and housing and husbandry of specimens. Further studies exploring these considerations are needed.

## Additional files

Additional file 1: PRISMA-P checklist. (DOCX 18 kb)

Additional file 2: SEARCH strategies. (DOCX 20 kb)

Additional file 3: Expanded eligibility criteria. (DOCX 17 kb)

## Abbreviations

CI: Confidence intervals
CPP: Cerebral perfusion pressure
eGOS: extended Glasgow outcome scale
GOS: Glasgow Outcome Scale
HSS: Hypertonic saline solutions
ICP: Intracranial pressure
mRS: modified Rankin scale
NMA: Network meta-analysis
NS: Normal saline
OR: Odds ratio
RCT: Randomized controlled trial
RL: Ringer’s lactate
RR: Relative risk ratio
