# Supplementary material for: Comparison of crystalloid resuscitation fluids for treatment of acute brain injury: a clinical and pre-clinical systematic review and network meta-analysis protocol
Source: Syst Rev. 2018 Aug 17;7:125. doi: 10.1186/s13643-018-0790-x (PMC6097326; doi:10.1186/s13643-018-0790-x)
Supplement: Supplementary file 2 — SEARCH strategies. (DOCX 20 kb) [file 13643_2018_790_MOESM2_ESM.docx]

**Additional file 2: SEARCH STRATEGIES**

## The following databases will be used to conduct our search strategy:

1. EMBASE Classic + Embase (1947 to present)
2. Epub Ahead of Print, In-Process & Other Non-Indexed Citations, Ovid MEDLINE(R) Daily and Ovid MEDLINE(R) (1946 to present)
3. Web of Science BIOSIS Previews (1926 to present)
4. EBM Reviews (incl Cochrane) (2005 to present)
5. Search of trial registers (clinicaltrials.gov) for ongoing and unpublished studies

**SAMPLE** **Search Strategies:**

**Database: Ovid MEDLINE(R) ALL <1946 to June 05, 2018>**

**Search Strategy:**

**--------------------------------------------------------------------------------**

1     exp Brain Injuries/ (61693)

2     brain injur*.tw. (55279)

3     head injur*.tw. (22311)

4     exp Stroke/ (114319)

5     exp Brain Ischemia/ (97628)

6     exp Cerebral Hemorrhage/ (31579)

7     exp "Intracranial Embolism and Thrombosis"/ (20196)

8     exp Subarachnoid Hemorrhage/ (19547)

9     ((Subarachnoid or intracranial or cerebral) adj2 Hemorrhag*).tw. (30761)

10     exp Craniocerebral Trauma/ (146078)

11     head trauma.tw. (7926)

12     exp Intracranial Pressure/ (14346)

13     ((brain or cerebral) adj2 ischemia).tw. (27351)

14     neurocritical care.tw. (950)

15     neuroICU.tw. (18)

16     Intensive care Units/ (46685)

17     or/1-16 (445714)

18     (ringer* adj2 lactate*).mp. (4508)

19     ringers solution.mp. (4732)

20     Hartmann$.mp. (4002)

21     plasmalyte.mp. (121)

22     plasma lyte.mp. (136)

23     isotonic solutions/ (8223)

24     balanced crystalloid$.tw. (157)

25     crystalloid solution$.tw. (774)

26     ringers acetate.mp. (268)

27     hypertonic solutions/ or saline solution, hypertonic/ (10848)

28     (hypertonic adj3 (saline or solution*)).tw. (8268)

29     hypertonicity.tw. (1901)

30     hyperosmolar.tw. (3024)

31     Sodium Chloride/ (56516)

32     sodium chloride.tw. (15664)

33     or/18-32 (97478)

34     17 and 33 (2056)

35     remove duplicates from 34 (2043)

**Database: Embase Classic+Embase <1947 to 2018 June 06>**

**Search Strategy:**

**--------------------------------------------------------------------------------**

1     exp *Brain Injuries/ (88817)

2     brain injur*.tw. (79142)

3     head injur*.tw. (30198)

4     exp *Stroke/ (71141)

5     exp *Brain Ischemia/ (80560)

6     exp *Cerebral Hemorrhage/ (49472)

7     exp *Cerebral Hemorrhage/ (49472)

8     exp *Intracranial pressure/ (6891)

9     *Craniocerebral trauma/ (17450)

10     ((brain or cerebral) adj2 ischemia).tw. (36974)

11     ((Subarachnoid or intracranial or cerebral) adj2 Hemorrhag*).tw. (44354)

12     neurocritical care.tw. (1746)

13     neuroICU.tw. (106)

14     head trauma.tw. (11282)

15     or/1-14 (365800)

16     (ringer* adj2 lactate*).mp. (9213)

17     ringers solution.mp. (6286)

18     (hartmann* adj2 solution).mp. (668)

19     plasmalyte.mp. (267)

20     plasma lyte.mp. (197)

21     balanced crystalloid*.tw. (252)

22     crystalloid solution*.tw. (1059)

23     ringers acetate.mp. (261)

24     isotonic solutions/ (5201)

25     (hypertonic adj3 (saline or solution*)).tw. (11699)

26     hypertonicity.tw. (2635)

27     hyperosmolar.tw. (4180)

28     hypertonic.tw. (20085)

29     *hypertonic solutions/ or *saline solution, hypertonic/ (34550)

30     or/16-29 (72935)

31     15 and 30 (1890)

**Database: Web of Science BIOSIS Previews <1926 to Present>**

**Search strategy:**

--------------------------------------------------------------------------------

TOPIC: (ringers lactate) OR TOPIC: (ringers acetate) OR TOPIC: (ringers solution) OR TOPIC: (lactated ringers) OR TOPIC: (hartmann's solution) OR TOPIC: (plasma lyte) OR TOPIC: (balanced crystalloid) OR TOPIC: (crystalloid solution) OR TOPIC: (hypertonic saline) OR TOPIC: (hypertonic solution) OR TOPIC: (hyperosmolar solution) OR TOPIC: (hypertonicity) OR TOPIC: (osmololar therapy) OR TOPIC: (sodium chloride) OR TOPIC: (sodium solution) OR TOPIC: (isotonic solution)

AND

TOPIC: (brain injur*) OR TOPIC: (stroke) OR TOPIC: (brain ischemia) OR TOPIC: (cerebral ischemia) OR TOPIC: (head injur*) OR TOPIC: (head trauma) OR TOPIC: (brain trauma) OR TOPIC: (brain contusion) OR TOPIC: (head contusion) OR TOPIC: (craniocerebral trauma) OR TOPIC: (subarachnoid hemorrhage) OR TOPIC: (intracranial hemorrhage) OR TOPIC: (cerebral hemorrhage) OR TOPIC: (intracranial embolism) OR TOPIC: (intracranial thrombosis) OR TOPIC: (close* head injur*)

**Database: Cochrane CENTRAL**

**Search strategy:**

--------------------------------------------------------------------------------

“ringers lactate” OR “ringers acetate” OR “ringers solution” OR “lactated ringers” OR “hartmann's solution” OR “plasma lyte” OR “balanced crystalloid” OR “crystalloid solution” OR "hypertonic" OR "hyperosmolar" OR "hypertonicity" OR "sodium solution" OR "osmol$ therapy" OR "sodium chloride"

AND

"brain injur*" or "brain contusion" or "closed head injur*" or "brain ischemia" or "cerebral ischemia" or "head injur*" or "intracranial hemorrhage" or "cerebral hemorrhage" or "head trauma" or MeSH descriptor: [Stroke] 1 tree(s) exploded or MeSH descriptor: [Brain Injuries] explode all trees or MeSH descriptor: [Craniocerebral Trauma] explode all trees or MeSH descriptor: [Subarachnoid Hemorrhage] 2 tree(s) exploded

**Database: clinicaltrials.gov**

**Search strategy:**

--------------------------------------------------------------------------------

(brain injury OR stroke OR cerebral ischemia OR head injury OR subarachnoid hemorrhage OR cerebral hemorrhage OR head trauma OR craniocerebral trauma) AND (ringers OR hartmanns solution OR plasma lyte OR crystalloid solutions OR hypertonic saline)
