# Supplementary material for: Comparison of crystalloid resuscitation fluids for treatment of acute brain injury: a clinical and pre-clinical systematic review and network meta-analysis protocol
Source: Syst Rev. 2018 Aug 17;7:125. doi: 10.1186/s13643-018-0790-x (PMC6097326; doi:10.1186/s13643-018-0790-x)
Supplement: Supplementary file 3 — Expanded eligibility criteria. (DOCX 17 kb) [file 13643_2018_790_MOESM3_ESM.docx]

**Additional file 3: ELIGIBILITY CRITERIA TABLE** **FOR THE SYSTEMATIC REVIEW**

**Table 1.1 Expanded eligibility criteria table for the systematic review**

| *Criteria* | *Description* |
| --- | --- |
| *Population* | We will include all studies examining acute brain injury (human and in-vivo animal brain injury models) within the first 7 days of therapy. We are targeting acute primary neurological diagnoses such as traumatic brain injury, stroke, hemorrhage, or post-neurosurgical care. Studies with mixed populations will be included if at least 50% of the population is composed of our target neurological population, or if a separate neurological subgroup is clearly presented within the study to allow for data extraction for these subgroups. In instances of lack of clarity, clarification with the corresponding author will be attempted. Studies evaluating participants without neurological diagnoses and/or only after the first 7 days post neurological injury will be excluded. |
| *Intervention* | The intervention of interest is the intravenous use of hypotonic crystalloid resuscitation fluids. These include Ringer’s Lactate, Hartmann’s or Plasma Lyte® fluids. These can be administered as bolus or maintenance infusions. There is no limit on dose or frequency of administration. |
| *Comparator(s)* | The main comparator of interest is an isotonic crystalloid resuscitation fluid, specifically Normal Saline (0.9%), which will serve as the prototypical control. Other crystalloid resuscitation fluids (ie: hypertonic saline (3-23.4%)) will also be included and act as a third challenger. These can be administered as bolus or maintenance infusions. There is no limit on dose or frequency of administration. We anticipate identifying studies in which the comparator may include a crystalloid fluid mixed with a colloid (ex: hydroxyethyl starch, gelatin, dextran). All such studies will be included; no study will be excluded based on type of comparator. |
| *Outcome(s)* | The primary outcomes of interest are:   1. Intracranial pressure (ICP) – mean, within group and across group mean difference (before and after administration) 2. Cerebral perfusion pressure (CPP) – mean, within group and across mean difference (before and after administration)   These primary outcomes were chosen as they are objective, relatively easily and frequently measured and have clinical sensibility in the possible biologic effects of these fluids. They are also measured in both clinical and pre-clinical studies.  Secondary outcomes include:   - Cerebral edema (as assessed on neuro imaging) - Serum electrolyte concentrations (Na+, Cl-) and osmolality - Brain electrolyte concentrations (Na+, Cl-) and osmolality - Clinical outcomes including modified Rankin Scale (mRS), Glasgow Outcome Scale (GOS), extended Glasgow Outcome Scale (eGOS) and mortality - Adverse events as defined by the study authors, including bradycardia, hypotension, or any other adverse event;   We will collect data on all parameters reported by authors to ensure this review is as inclusive as possible. No studies will be excluded based on lack of reported outcomes. |
| *Study Design* | We will include all completed English language publications reporting the intravenous use of crystalloid fluids in acute brain injury in our quantitative review, including randomized controlled trials, quasi-randomized trials, and retrospective and prospective cohort studies that include a control group for comparison. Both clinical and pre-clinical studies (in-vivo animal brain injury models) will be included. There will be no date restrictions applied in any of the searches. In-progress studies identified from the clinicaltrials.gov registry and CENTRAL Cochrane will be included in a qualitative analysis. Letters to the editor, case reports, case series, editorial reviews and guidelines will be excluded. |
